# Diabetes mellitus in pregnancy across Canada

**DOI:** 10.1186/s12884-024-06534-8

**Published:** 2024-05-07

**Authors:** Chantal RM Nelson, Susie Dzakpasu, Aideen M. Moore, Elizabeth K. Darling, Wesley Edwards, Phil Murphy, Heather Scott, Michiel Van Den Hof, Joel G. Ray

**Affiliations:** 1 Maternal and Infant Health Section, Centre for Surveillance and Applied Research, Public Health Agency of Canada, 785 Carling Ave, Ottawa, ON Canada; 2 Department of Paediatrics, The Hospital for Sick Children and University of Toronto, Toronto, ON Canada; 3 McMaster Midwifery Research Centre, Department of Obstetrics and Gynecology, McMaster University, Hamilton, ON Canada; 4 Department of Anesthesia and Pain Medicine, Faculty of Medicine, University of Ottawa, Ottawa, ON Canada; 5 Newfoundland and Labrador Health Services CA, St. John’s, Newfoundland and Labrador, St. John’s, Canada; 6 Department of Obstetrics and Gynaecology, Dalhousie University, IWK Health Centre, Halifax, NS Canada; 7 Departments of Medicine, Health Policy Management and Evaluation, and Obstetrics and Gynaecology, St Michael’s Hospital, University of Toronto, Toronto, ON Canada

**Keywords:** Pre-existing diabetes, Type 2 diabetes, Gestational diabetes, pregnancy, trend, Canada

## Abstract

**Background:**

Contemporary estimates of diabetes mellitus (DM) rates in pregnancy are lacking in Canada. Accordingly, this study examined trends in the rates of type 1 (T1DM), type 2 (T2DM) and gestational (GDM) DM in Canada over a 15-year period, and selected adverse pregnancy outcomes.

**Methods:**

This study used repeated cross-sectional data from the Canadian Institute of Health Information (CIHI) hospitalization discharge abstract database (DAD). Maternal delivery records were linked to their respective birth records from 2006 to 2019. The prevalence of T1DM, T2DM and GDM were calculated, including relative changes over time, assessed by a Cochrane-Armitage test. Also assessed were differences between provinces and territories in the prevalence of DM.

**Results:**

Over the 15-year study period, comprising 4,320,778 hospital deliveries in Canada, there was a statistically significant increase in the prevalence of GDM and T1DM and T2DM. Compared to pregnancies without DM, all pregnancies with any form of DM had higher rates of hypertension and Caesarian delivery, and also adverse infant outcomes, including major congenital anomalies, preterm birth and large-for-gestational age birthweight.

**Conclusion:**

Among 4.3 million pregnancies in Canada, there has been a rise in the prevalence of DM. T2DM and GDM are expected to increase further as more overweight women conceive in Canada.

**Supplementary Information:**

The online version contains supplementary material available at 10.1186/s12884-024-06534-8.

## Background

Diabetes mellitus (DM) during pregnancy is an important health indicator, as it is associated with an increased risk of adverse outcomes for both the birthing parent and their infant(s). Pre-existing type 1 (T1DM) and type 2 (T2DM) DM in pregnancy are known to be associated with an increased risk of adverse perinatal outcomes, such as perinatal mortality, preterm birth, and congenital anomalies [1, 2]. Gestational DM (GDM) is known to increase the risk of macrosomia, intrauterine fetal death, preterm birth, congenital anomalies, and respiratory distress syndrome [3, 4]. Furthermore, a person who develops GDM has an increased risk of developing T2DM or impaired glucose tolerance in the years following pregnancy [5].

Recent estimates show that the prevalence of T2DM and GDM is increasing globally [6, 7]. A rise in T1DM is being documented internationally in younger populations [8–10]. Although there are national reports on DM during pregnancy [11, 12], few distinguish between pre-existing DM and GDM. This paper explored the temporal trends of pregnancies with T1DM, T2DM and GDM in Canada using hospital record data over a 15-year period.

## Methods

### Study design and setting

This retrospective repeated cross-sectional study was conducted using hospitalization data from the Canadian Institute of Health Information (CIHI) acute-care discharge abstract database (DAD) over a 15-year period, from 2005 to 2019. The DAD captures administrative, clinical and demographic information on hospital discharges in Canada [13]. The DAD has been shown to have high sensitivity and specificity on many maternal and infant health variables [14]. Almost all persons in Canada give birth in hospitals (98%); [15] therefore, the CIHI-DAD captures most deliveries in Canada and is the largest Canadian data source for diagnoses of maternal health conditions, such as T1DM and T2DM and/or GDM. Data are received directly from acute care facilities or their respective health/regional authority or ministry/department of health. Facilities in all provinces and territories except Quebec are required to report to CIHI [16]. 

### Participants

Any woman aged 15–54 years who delivered a liveborn or stillborn baby in a Canadian hospital (except in the province of Quebec) between the 2005/2006 to 2019/2020 fiscal years was eligible for inclusion in the study. The DAD was used to identify in maternal records of those who were admitted to hospital to deliver a baby using International Statistical Classification of Diseases and Related Health Problems, Tenth Revision, Canadian (ICD-10-CA) codes. Records with an ICD-10-CA code of O10-O16, O21-O29, O30-O37, O40-O46, O48, O60-O75, O85-O92, O95 or O98-O99 with a ‘1’ or ‘2’ coded at the 6th digit, or Z37 coded in any position were used to identify delivery records. Abortive procedures were excluded. The standard maternal population includes persons aged 10–54; however, we excluded persons aged 10–14 years due to small cell counts of 1223 deliveries. The final study sample consisted of 4,320,778 hospital deliveries.

### Outcomes

Diabetes during pregnancy was defined using ICD-10-CA codes as follows. For fiscal years 2005/06 and 2006/07 records with ‘O240’ or ‘E10’ were coded as pre-existing T1DM; records with ‘O241’ or ‘E11’ were coded as pre-existing T2DM; and records with ’O244’ were coded as GDM. Due to changes in coding practices, from 2007/08 onwards, records with ‘O245’ or ‘E10’ were coded as T1DM; records with ‘O246’ or ‘E11’ were coded as T2DM; and records with ‘O248’ were coded as GDM. Unspecified DM or DM due to malnutrition were excluded. Supplemental Table 1 has the complete list of ICD-10-CA codes.

Maternal characteristics include age at delivery (grouped by 5-year intervals), pregnancy type (singleton or multiple), rural or urban residence—determined by first three digits of postal code, hypertension during pregnancy, gestational age at birth (< 32 weeks, 32–36 weeks, 37–42 weeks), type of preterm birth (spontaneous or provider-initiated), type of delivery (vaginal, c-section, induction), parity (first time mother or previous delivery) and outcome of delivery (stillbirth or livebirth). Maternal records were linked to birth records to assess if a major congenital anomaly was present as defined by the Canadian Congenital Anomalies Surveillance System [17], if the infant had any birth trauma (using ICD-10-CA codes P10-P15), and to determine birth weight percentiles, expressed as small- (SGA) or large- (LGA) for gestational age birthweight based on Kramer et al.’s methodology [18]. The proportion of missing data was 21% for parity between 2005 and 2015, after which it was < 0.1%, 0.4% for rural/urban residence and 0.6% SGA/LGA (due to missing birthweight). The data were complete for all clinical interventions (type of delivery, preterm birth status), diagnoses and birth outcomes. The study population distribution or the prevalence rates were the same with or without missing data.

### Statistical analysis

We calculated the prevalence of DM during pregnancy using deliveries in hospital as the denominator, and any person with a diagnosis of T1DM, T2DM or GDM found on the delivery record as the respective numerators and expressed as a proportion per 1000 deliveries. We calculated temporal trend tests using the Cochrane-Armitage test. We also calculated relative percent changes between 2019 and 2005. Maternal DM rates were also stratified by fiscal year (April 1-March 31).

We described the adverse pregnancy outcomes stratified by DM type and those without DM. Chi-squared tests and *p* < 0.05 were used to determine if the differences were statistically significant.

We stratified gestational DM by parity to show differences in rates among women who have had a previous pregnancy versus nulliparous mothers. To compensate for the missing parity data between 2005 and 2014, we imputed the missing values using a linear regression approach. We compared the distribution of gestational DM to those with and missing parity information, and as the distribution was similar, it was determined that the data was missing at random [19]. We included maternal age, province of residence, gestational diabetes, mode of delivery and pregnancy type as covariates that may influence the missing values as part of the regression model to minimize bias [19]. The distribution of data were similar in both the imputed and original data. We included the imputed data to populate Tables 1 and 2. All other analysis were done on the original dataset as the slope of change in gestational diabetes remained consistent over time, with or without parity information.

**Table 1:** Maternal characteristics and select pregnancy and newborn measures by type of diabetes mellitus (DM) among 4,320,778 hospital deliveries in Canada, 2005–2019. Excluded is the province of Quebec. All data are presented as a number (%). † Data are based on linked records

Characteristic	Type 1 DM (*N* = 12,172)	Type 2 DM (*N* = 21,782)	Gestational DM (*N* = 286,897)	No DM (*N* = 4,001, 876)
Age group, y				
*15–19*	319 (2.6)	274 (1.3)	2593 (0.9)	150,082 (3.8)
*20–24*	1743 (14.3)	1375 (6.3)	17,128 (6.0)	573,811 (14.3)
*25–29*	3749 (30.8)	3997 (18.4)	59,362 (20.7)	1163,526 (29.1)
*30–34*	4046 (33.2)	7147 (32.8)	105,268 (36.7)	1,334,637 (33.4)
*35–39*	1970 (16.2)	6525 (30.0)	78,516 (27.4)	649,493 (16.2)
*40–44*	330 (2.7)	2264 (10.4)	22,175 (7.7)	123,142 (3.1)
*45+*	15 (0.1)	200 (0.9)	1855 (0.7)	7183 (0.2)
Pregnancy type				
*Singleton*	11,840 (97.3)	20,662 (94.9)	275,787 (96.1)	3,891,225 (97.2)
*Multiple*	332 (2.7)	1120 (5.1)	11,110 (3.9)	110,651 (2.8)
Parity				
*Nulliparous*	5,752 (47.3)	6,828 (31.3)	109,842 (38.3)	1,708,499 (42.7)
*Parous*	6,420 (52.7)	14,954 (68.7)	177,055 (61.7)	2,293,377 (57.3)
Area of residence^†^				
*Rural*	2342 (19.2)	4955 (22.8)	36,340(12.7)	713,721 (17.8)
*Urban*	9830 (80.8)	16,827 (77.3)	250,557 (87.3)	3,288,155 (82.2)
Hypertension	3087 (25.4)	6019 (27.6)	334,833 (12.2)	242,071 (6.0)
Gestational age, weeks				
*< 32*	374 (3.1)	721 (3.3)	3510 (1.2)	39,438 (1.0)
*32–36*	3156 (25.9)	4105 (18.8)	26,496 (9.2)	228,197 (5.7)
*37–42*	8642 (70.1)	16,956 (77.8)	256,891 (89.5)	3,734,241 (93.3)
Type of preterm birth				
*Spontaneous*	1902 (53.8)	2382 (49.7)	17,967 (59.9)	186,698 (69.8)
*Provider-initiated*	1491 (12.2.)	2188 (50.3)	9933 (33.1)	63,729 (23.8)
Mode of birth				
*Vaginal*	4292 (35.3)	9433 (43.3)	168,101 (58.6)	2,784,273 (69.6)
*Caesarean*	7880 (64.7)	12,349 (55.7)	118,796 (41.1)	1,217,603 (30.4)
Induction of labour	4996 (41.1)	9685 (44.5)	112,242 (39.1)	961,185 (24.0)
Stillbirth	235 (1.9)	453 (1.8)	1094 (0.4)	22,749 (0.6)
Newborn weight^†^				
*Small for gestational age < 10th centile*	318 (2.6)	1457 (6.7)	23,370 (6.7)	325,513(8.2)
*Large for gestational age > 90th centile*	4935 (41.0))	5973 (27.8)	37,900 (13.3)	334,756 (8.4)
Missing				
Major congenital anomaly^†^	291 (2.4)	584 (2.7)	4007 (1.4)	46,673 (1.2)
Birth trauma^†^	428 (3.5)	572 (2.6)	4754 (1.7)	65,535(1.6)

**Table 2:** Annual temporal trends in gestational (DM) by parity, among 4,320,778 hospital deliveries in Canada, 2005–2019. Excluded is the province of Quebec. All data are presented as a rate per 1000

	Year																
Parity	2005	2006	2007	2008	2009	2010	2011	2012	2013	2014	2015	2016	2017	2018	2019	Trend *p*-value	% relative change^a^
Gestational DM																	
*Nulliparous*	35.0	38.3	38.9	41.9	46.1	47.9	51.0	55.7	60.0	65.0	69.0	77.9	82.6	89.1	98.3	< 0.001	180.7
*Parous*	45.5	49.0	49.1	53.4	56.9	59.0	63.5	67.4	70.1	75.6	80.1	90.4	96.7	100.4	108.7	< 0.001	138.9

^a^Percent relative change comparing the rate in 2019 vs. the rate in 2005

To examine geographic differences, we calculated the prevalence of DM during pregnancy by DM type and province, in five-year intervals. Stratified by DM type, we also calculated prevalence using Ontario as the reference as it contains the largest proportion of deliveries in Canada. In 2011, British Columbia (BC) universally adopted the ‘alternative’ 1 step approach to screen for GDM, which has lower diagnostic thresholds than the ‘preferred’ two-step approach. The preferred approach uses a standardized non-fasting 50-g glucose challenge screening test (GCT) with plasma glucose (PG) measured 1 h later. If the value of the GCT is 7.8–11.0, a 2-hour 75-g oral glucose tolerance test with fasting is performed. GDM is diagnosed if any of the criteria are met: FPG ≥ 5.3 mmol/L, 1-h PG ≥ 10.6 mmol/L or 2-h PG ≥ 9.0 mmol/L. The alternative 1-step approach uses a standardized 2-hour 75-g oral glucose tolerance test with fasting plasma glucose, 1-hour plasma glucose (PG), and 2-hour PG. GDM is diagnosed if any of the criteria are met: FPG ≥ 5.1 mmol/L, 1-h PG ≥ 10.0 mmol/L or 2-h PG ≥ 8.5 mmol/L. This change in practice resulted in a higher number of GDM cases. As BC’s data likely amplifies the national rates due to their testing practices, national estimates are presented with and without BC.

Any estimate with a count < 5 in the numerator was excluded for privacy reasons. Data were analysed using SAS EG version 7.1 and graphs were produced using Microsoft Excel v2016.

Ethics approval was not required as this study was based on anonymized data and conducted under the surveillance mandate of the Public Health Agency of Canada.

## Results

**Fig. 1:**
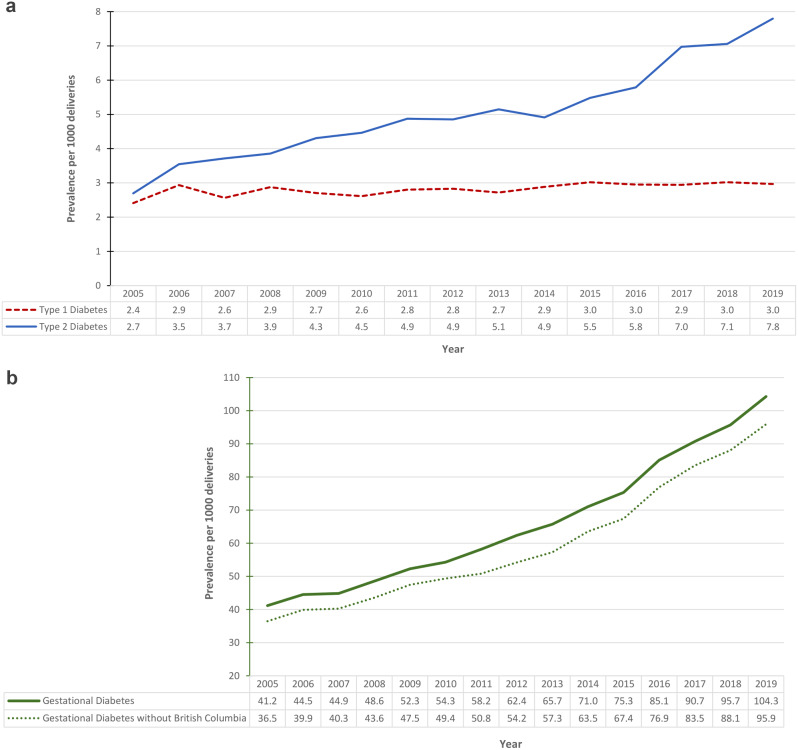
(**a**) Annual temporal trends in type 1 and type 2 diabetes mellitus (DM) among hospital deliveries in Canada, 2005–2019. Excluded is the province of Quebec. (**b**) Annual temporal trends in gestational diabetes mellitus (GDM) among hospital deliveries in Canada, 2005–2019. Excluded is the province of Quebec. Furthermore, the lower dashed line does not include data from the province of British Columbia

The study included 4,320,778 hospital deliveries in Canada over the 15-year study period. Across this period there was a statistically significant increase in T1DM and T2DM (Fig. 1a) as well as GDM (Fig. 1b). The relative increase in T2DM was 189% (2.7 per 1000 deliveries to 7.8 per 1000 deliveries), 25% (2.4 per 1000 deliveries to 3.0 per 1000 deliveries) for T1DM, 162.7% for GDM (36.5 per 1000 deliveries to 95.9 per 1000 deliveries without BC), and 153% (41.2 per 1000 deliveries to 104.3 per 1000 deliveries, including BC).

The prevalence of adverse pregnancy outcomes varied by DM status. Persons with T1DM and T2DM had a higher prevalence of adverse infant outcomes including major congenital anomalies, preterm birth, LGA and stillbirth compared to those without DM. Persons with GDM had a higher prevalence of preterm birth, caesarean section, induction or LGA compared to those without DM. Persons with GDM also had slightly higher rates of congenital anomalies and birth trauma, but lower rates of stillbirth than those without DM. All persons who had any DM had a higher prevalence of hypertension than those without DM. See Table 1.

The largest increases in both GDM and T1DM and T2DM were found among the youngest population, aged 15–19 years old. For this age group, between 2005 and 2019, there was a relative increase of 260% for GDM (including BC), a relative increase of 291% (without BC) a relative increase of 330% for T2DM, and a relative increase of 92.9% for T1DM (see Table 3).

**Table 3:** Annual temporal trends by type of diabetes mellitus (DM) and age groups, among 4,320,778 hospital deliveries in Canada, 2005–2019. Excluded is the province of Quebec. All data are presented as a rate per 1000

	Year																
Age group	2005	2006	2007	2008	2009	2010	2011	2012	2013	2014	2015	2016	2017	2018	2019	Trend *p*-value	% relative change^a^
Type 1 DM																	
*15–19 y*	1.4	1.9	1.8	1.6	2.1	2.3	2.0	2.6	2.4	2.2	1.7	3.4	1.6	2.9	2.7	< 0.001	92.9
*20–24 y*	2.2	2.8	2.4	3.0	2.6	2.4	2.9	3.0	3.2	3.5	3.6	3.2	3.4	3.4	3.7	< 0.001	68.2
*25–29 y*	2.3	3.0	2.8	2.9	3.5	2.7	3.1	3.2	3.0	3.0	3.0	3.2	3.4	3.2	3.3	0.03	43.5
*30–34 y*	2.6	3.1	2.6	3.0	2.4	2.5	2.7	2.8	2.6	2.8	3.0	2.6	3.0	3.2	2.9	0.04	11.5
*35–39 y*	2.5	3.1	2.7	2.9	2.2	3.0	2.8	2.4	2.3	2.8	2.9	3.0	2.4	2.4	2.6	0.81	4.0
*40–44 y*	2.8	2.7	2.0	2.2	2.1	2.5	2.0	2.5	2.5	2.7	2.8	2.0	1.5	2.8	1.4	0.07	-50.0
*45 + y*	NA	NA	NA	NA	NA	NA	NA	NA	NA	NA	NA	NA	NA	NA	NA	NA	NA
***Type 2 DM***																	
*15–19 y*	1.0	0.9	0.6	1.0	1.1	1.9	1.6	2.2	1.7	2.2	2.7	3.2	4.1	2.5	4.3	< 0.001	330.0
*20–24 y*	1.8	1.9	1.8	1.5	1.9	1.9	2.2	2.1	2.4	2.4	2.5	2.7	3.7	3.4	4.1	< 0.001	127.8
*25–29 y*	1.8	2.6	2.8	2.4	3.2	3.0	3.3	3.1	3.4	3.0	3.4	3.6	4.2	4.4	4.6	< 0.001	155.6
*30–34 y*	2.8	3.7	3.9	4.2	4.5	4.9	4.6	5.1	4.8	4.8	5.2	5.2	6.3	6.1	6.7	< 0.001	139.3
*35–39 y*	4.4	6.4	6.7	7.3	7.6	7.9	9.2	8.1	8.7	8.6	9.3	9.8	11.2	11.8	12.7	< 0.001	188.6
*40–44 y*	8.0	10.2	10.9	12.1	11.6	11.6	14.3	13.7	17.8	14.8	16.6	18.6	19.9	20.5	22.9	< 0.001	186.3
*45 + y*	NA	NA	NA	NA	NA	NA	NA	NA	NA	NA	NA	NA	NA	NA	NA	NA	NA
***Gestational DM with BC***																	
*15–19 y*	10.6	12.6	11.2	12.3	12.6	14.4	16.7	18.4	18.3	20.8	22.2	23.5	25.6	25.6	38.2	< 0.001	260.4
*20–24 y*	18.9	20.9	20.0	21.6	21.7	24.5	26.2	27.8	29.5	33.0	32.2	38.2	42.9	47.4	49.4	< 0.001	161.4
*25–29 y*	33.5	34.0	34.8	37.2	40.1	40.5	42.7	44.7	47.8	49.7	53.2	60.1	64.8	67.5	76.4	< 0.001	128.1
*30–34 y*	45.6	50.7	51.8	54.6	58.0	59.8	64.0	68.6	72.1	76.2	80.8	89.2	93.3	97.4	104.8	< 0.001	129.8
*35–39 y*	67.1	74.3	73.0	81.2	87.5	91.0	94.5	102.1	102.0	111.8	116.8	131.4	135.3	141.7	149.0	< 0.001	122.1
*40–44 y*	101.0	104.7	106.0	199.2	133.5	121.8	135.1	136.8	148.9	162.8	162.3	180.6	190.5	188.3	203.6	< 0.001	101.6
*45 + y*	106.3	151.4	135.1	159.1	153.5	168.0	173.7	177.3	200.0	224.8	243.5	216.1	239.5	239.8	253.1	< 0.001	138.1
***Gestational DM without BC***																	
*15–19 y*	9.9	12.1	11.4	12.8	12.9	14.7	15.6	16.3	18.0	19.7	21.4	22.7	23.9	27.3	37.8	< 0.001	290.9
*20–24 y*	17.9	19.2	19.4	20.3	20.8	23.1	24.0	24.9	27.1	29.9	29.8	35.7	40.2	44.6	46.3	< 0.001	158.7
*25–29 y*	29.9	30.6	31.3	33.2	37.3	37.0	38.1	39.1	41.5	45.3	47.6	54.5	60.1	62.4	70.1	< 0.001	134.4
*30–34 y*	40.7	45.7	46.8	49.5	52.3	54.7	55.6	59.3	62.7	69.0	72.9	81.4	87.2	90.6	97.2	< 0.001	138.8
*35–39 y*	66.0	68.1	66.4	73.0	79.8	83.4	83.6	92.0	90.7	102.1	107.1	120.4	125.5	131.7	138.3	< 0.001	109.5
*40–44 y*	87.3	92.8	93.1	108.0	123.4	113.7	121.3	117.9	133.5	145.0	145.3	165.6	176.9	174.3	193.4	< 0.001	121.5
*45 + y*	98.9	140.5	134.3	158.2	149.2	150.2	158.6	168.3	202.2	185.9	214.7	217.9	215.8	221.9	227.7	< 0.001	130.2

^a^Percent relative change comparing the rate in 2019 vs. the rate in 2005

NA Suppressed due to small cell counts < 5

Mothers who have had a previous pregnancy had an overall higher prevalence of gestational DM compared to first time mothers, however the relative increase in GDM was highest among nulliparous mothers (180%) compared to parous mothers (136%) (see Table 2).

**Fig. 2:**
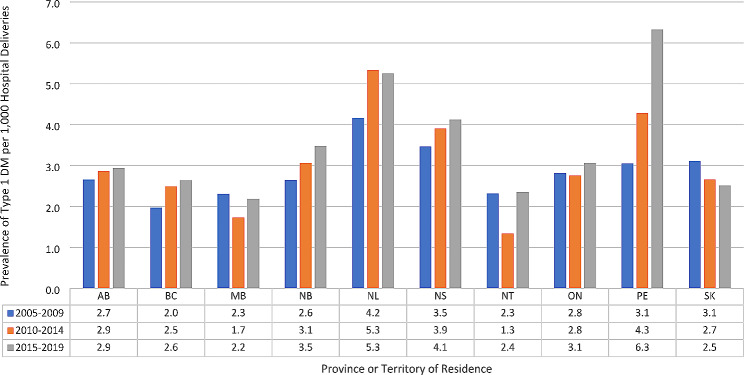
Prevalence of type 1 diabetes mellitus (DM) by five-year Intervals, by Canadian province, 2005–2019 Excluded is the province of Quebec. Furthermore, as the Yukon Territory and Nunavut each had cell counts < 5, they are not included in this figure Abbreviations: AB-Alberta, BC- British Columbia, MB-Manitoba, NB-New Brunswick, NL-Newfoundland and Labrador, NS-Nova Scotia, NT-Northwest Territories, ON- Ontario, PE- Prince Edward Island, SK-Saskatchewan

**Fig. 3:**
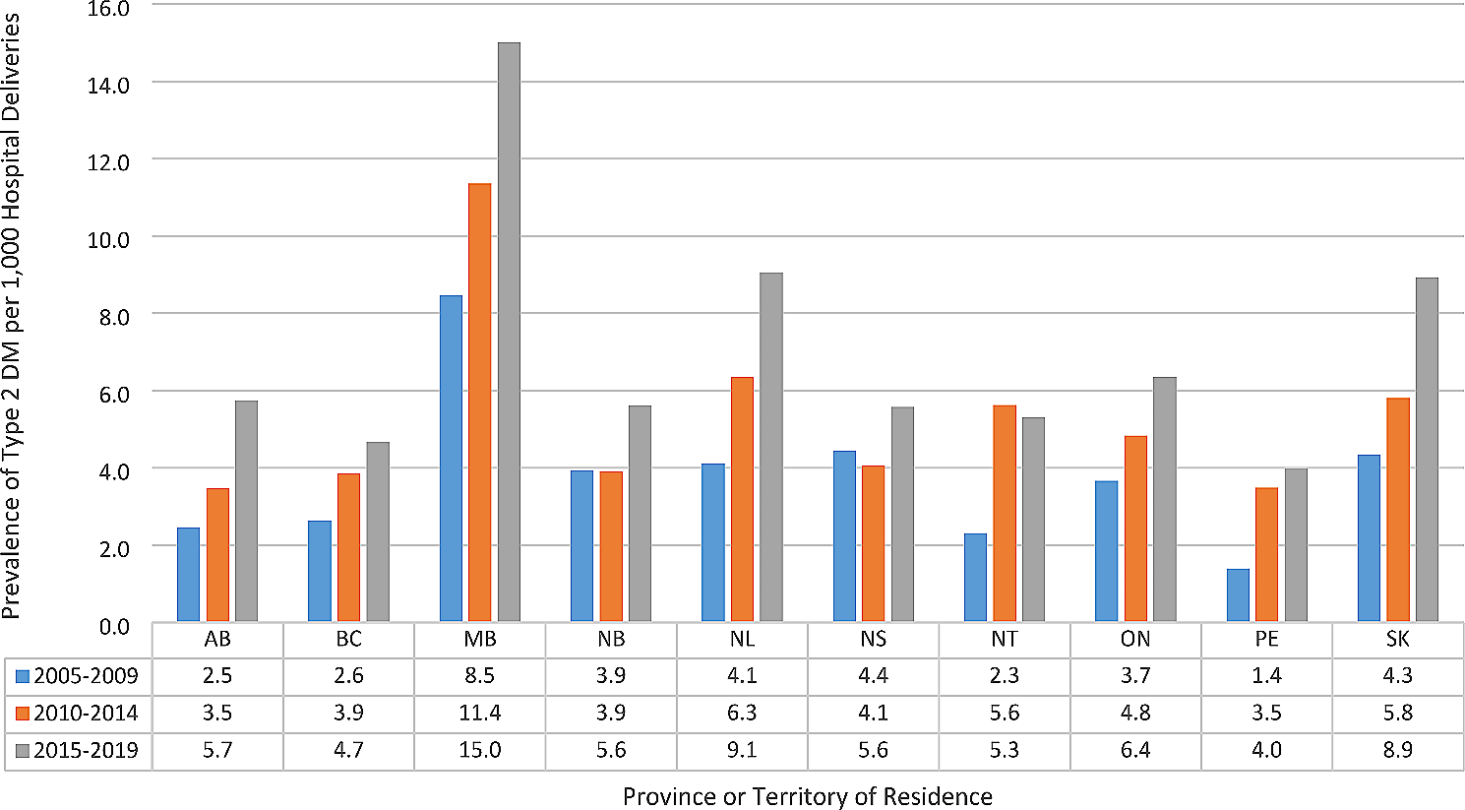
Prevalence of type 2 diabetes mellitus (DM) by five-year Intervals, by Canadian province or territory, 2005–2019 Excluded is the province of Quebec Yukon Territories and Nunavut had cell counts < 5 and thus, are omitted Abbreviations: AB-Alberta, BC- British Columbia, MB-Manitoba, NB-New Brunswick, NL-Newfoundland and Labrador, NS-Nova Scotia, NT-Northwest Territories, ON- Ontario, PE- Prince Edward Island, SK-Saskatchewan

**Fig. 4:**
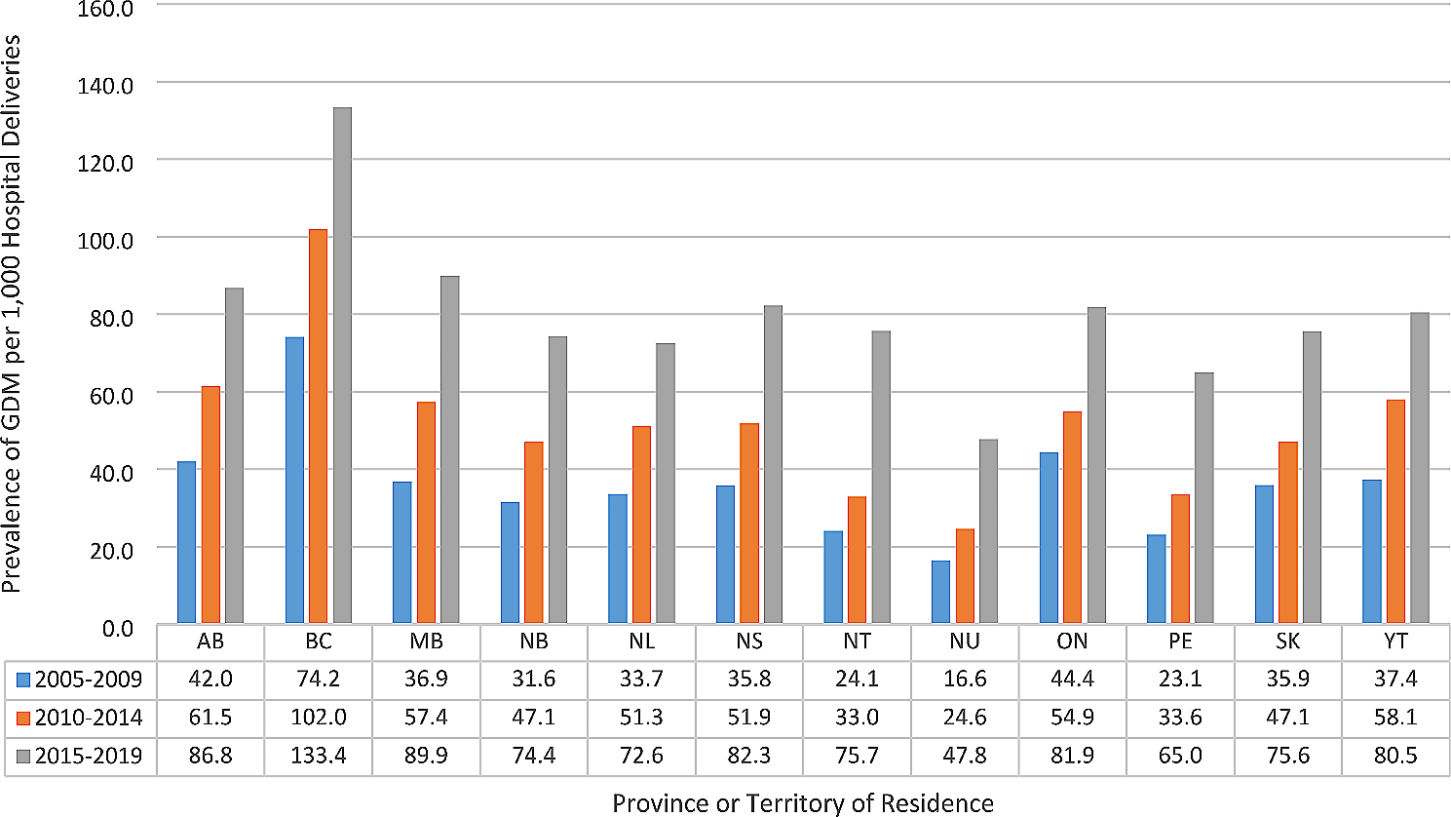
Prevalence of gestational diabetes mellitus (GDM) by five-year Intervals, by Canadian province or territory, 2005–2019 Excluded is the province of Quebec Abbreviations: AB-Alberta, BC- British Columbia, MB-Manitoba, NB-New Brunswick, NL-Newfoundland and Labrador, NS-Nova Scotia, NT-Northwest Territories, NU-Nunavut, ON- Ontario, PE- Prince Edward Island, SK-Saskatchewan, YT-Yukon Territories

The geographical distribution of DM varied across Canada (Table 4). The highest proportion of GDM cases were found in Western Canada, with British Columbia having the highest rate of 104.3 per 1000 deliveries followed by Alberta with 64.2 per 1000 deliveries. The fewest cases were reported in Nunavut, with a rate of 30.7 per 1000 deliveries. T2DM showed a very different distribution pattern, with the highest reported cases in Manitoba (11.8 per 1000 deliveries), followed by Saskatchewan (6.6 per 1000 deliveries) and the lowest prevalence was seen in Nunavut (1.3 per 1000 deliveries). T1DM was highest in Atlantic Canada, with Newfoundland and Labrador having the highest rate of 4.9 per 1000 deliveries, followed by Prince Edward Island at 4.4 per 1000 deliveries and the lowest prevalence was found in the Territories (2.0 per 1000 deliveries). T1DM and T2DM and GDM increased across all jurisdictions, except Nunavut, where this was only true for GDM. See Figs. 2, 3 and 4.

**Table 4:** Prevalence and prevalence ratios of diabetes mellitus (DM) across Canada’s jurisdictions, by DM type, among hospital deliveries in Canada, 2005–2019. Excluded is the province of Quebec. All data are presented as a rate per 1000. Ontario serves as the reference group, as it contains the most births among all jurisdictions

	Type 1 DM		Type 2 DM		Gestational DM	
Jurisdiction	Prevalence per 1000 hospital deliveries (95% CI)	Prevalence ratio (95% CI)	Prevalence per 1000 hospital deliveries (95% CI)	Prevalence ratio (95% CI)	Prevalence per 1000 hospital deliveries (95% CI)	Prevalence ratio (95% CI)
British Columbia	2.4 (2.3–2.5)	0.8 (0.8–0.9)	3.8 (3.6–3.9)	0.8 (0.7–0.8)	104.3 (103.1-105.1)	1.7 (1.7–1.7)
Alberta	2.8 (2.7-3.0)	1.0 (0.9-1.0)	3.9 (3.8–4.1)	0.8 (0.8–0.8)	64.2 (63.6–64.7)	1.1 (1.1–1.1)
Saskatchewan	2.7 (2.5-3.0)	0.9 (0.9-1.0)	6.6 (6.2–6.9)	1.3 (1.3–1.4)	54.5 (53.5–66.6)	0.9 (0.9–0.9)
Manitoba	2.1 (1.9–2.3)	0.7 (0.7–0.8)	11.8 (11.4–12.2)	2.4 (2.3–2.5)	62.8 (61.8–63.8)	1.0 (1.0-1.1)
***Ontario***	***2.9 (2.8-3.0)***	***1.00 (ref.)***	***4.9 (4.8-5.0)***	***1.00 (ref.)***	***60.0 (59.7–60.3)***	***1.00 (ref.)***
New Brunswick	3.1 (2.7–3.4)	1.1 (0.9–1.2)	4.5 (4.1–4.9)	0.9 (0.8-1.0)	50.4 (49.0-51.8)	0.8 (0.8–0.9)
Nova Scotia	3.8 (3.5–4.2)	1.3 (1.2–1.4)	4.7 (4.3–5.1)	0.9 (0.9-1.0)	56.0 (54.7–57.3)	0.9 (0.9-1.0)
Prince Edward Island	4.4 (3.5–5.4)	1.5 (1.2–1.9)	2.8 (2.1–3.7)	0.6 (0.4–0.7)	38.9 (36.2–41.7)	0.6 (0.6–0.7)
Newfoundland and Labrador	4.9 (4.4–5.5)	1.7 (1.5–1.9)	6.4 (5.8–7.1)	1.3 (1.2–1.4)	51.9 (50.2–53.7)	0.9 (0.8–0.9)
Northwest Territories	2.0 (1.2-3.0)	0.7 (0.4–1.1)	4.5 (3.3-6.0)	0.9 (0.7–1.2)	44.6 (40.6–48.9)	0.7 (0.7–0.8)
Yukon	2.2 (1.2–3.7)	0.8 (0.4–1.3)	3.2 (1.9-5.0)	0.6 (0.4-1.0)	60.0 (53.9–66.6)	1.0 (0.9–1.1)
Nunavut	NA	NA	1.3 (0.8–2.2)	0.3 (0.2–0.4)	30.7 (27.6–34.0)	0.5 (0.5–0.6)
***Canada combined, excluding Quebec***	***2.8 (2.8–2.9)***	***--***	***5.0 (5.0-5.1)***	***--***	***66.4 (66.2–66.6)*** ^***a***^ ***59.8 (59.0-60.6)*** ^***b***^	***--***

^a^Includes British Columbia within the overall Canadian prevalence estimate

^b^Omits British Columbia from the overall Canadian prevalence estimate

NA Suppressed due to small cell counts < 5

## Discussion

Over a 15-year period, there has been an increase in T1DM, T2DM and GDM. This is consistent with recent international data [20–22]. There was a positive relation between DM and maternal age for both T2DM and GDM, with a higher prevalence seen with each increase in age groups. Despite having the lowest rates of DM overall, the largest increases over the fifteen-year study period were found among persons aged 15–19, for GDM, T2DM and T1DM.

Estimates of the population-level prevalence of T1DM in pregnancies are limited, as most literature reports by combining ‘pre-existing’ DM types or involving smaller clinical study samples. Where data are available, our estimates show comparable results (0.3% had T1DM in our study). Four large studies in Scotland (15-year study period), the UK (16 years), the US (18 years) and Sweden (15 years) found that approximately 0.4%, 0.4%, 0.2% and 0.5% of pregnancies had T1DM at the end of the study period, respectively [20, 22–24]. In all studies, an increase in rates was seen over time which is consistent with our study findings. A US study among a cohort of youth less than 18 years of age (non-pregnant population) is projecting a tripling of T1DM and T2DM rates by 2050 [8]. A study in the Pima Indian population over a 30-year period found that increasing weight and increasing frequency of exposure to DM in utero accounted for most of the increase in the DM prevalence in Pima Indian children [25]. The increase in the prevalence of DM in our study is worrying as this increase may have an impact on DM risk among exposed offspring [26].

Established risk factors for developing T2DM or GDM are similar and include pre-pregnancy obesity, advanced maternal age, a positive family history of DM, and non-white ethnicity [2, 5]. Further, women who had GDM in one pregnancy may be at higher risk for GDM in a subsequent pregnancy [27, 28]. As our findings come from administrative data, it was not possible to examine most known individual-level risk factors, thus we cannot establish which risk factor(s) may most explain the increasing trend. We were able to observe a higher prevalence of gestational DM among parous women, though this does not appear to drive the increase over time, as the rate of change over time is consistent with nulliparous mothers. In large part, the increasing trend of an obesity among Canadian adults [29] may explain the increased rates of DM seen in this study. Additionally, increased trends may be due to possible generational epigenetic effects of obesity through maternal *in-utero* exposure to hyperglycemia [25]. As rates of GDM continue to increase, continued surveillance is important as persons who have GDM have a 35–60% chance of developing T2DM following their pregnancy, have higher risk for GDM in subsequent a pregnancy, and higher risk of adverse infants outcomes [3, 5, 6, 30].

Both Diabetes Canada [5] and the Society of Obstetricians and Gynaecologists of Canada (SOGC) [31] intermittently publish clinical guidelines for GDM screening. The recommendations for GDM screening have varied between iterations of those guidelines, especially following the publication of the Hyperglycemia and Adverse Pregnancy Outcome (HAPO) study [32]However, lack of a consensus on which is the optimal screening method continues, resulting in differing estimates of GDM prevalence in Canada. For example, historically British Columbia has had a higher prevalence of GDM than all provinces and territories [33]. In 2011, the province of British Columbia universally adhered to the alternate one-step screening approach for GDM [34], which further increased the prevalence of GDM in that province. Due to the potentially exaggerated increase of cases in British Columbia, national estimates were presented herein that included and excluded data from that province.

Diabetes status was ascertained using ICD-10-CA codes identified in the maternal delivery record. The use of ICD-10-CA coding appears to be a reliable and valid way to identify both T1DM and T2DM, and also GDM. A study comparing ICD-10 coding to medical records for persons who were delivered in hospital between 2016 and 2018 found that for GDM, ICD-10 codes had a high negative predictive value > 99% and a high specificity > 99% [35]. For pre-existing DM, the sensitivity was 85.9% (95% CI 78.8 to 93.0) and the positive predictive value was 91% (95% CI 85 to 97) [35]. Similarly, a validation study conducted in Alberta found that ICD-10 codes for GDM in administrative databases can be used to reliably estimate the burden of the disease at the population level and that delivery record codes are likely more accurate as these codes are included at the end of the hospital stay and have likely been verified [36]. While the diagnostic thresholds for assessing GDM have remained consistent in Canada during the study period [8, 37], there is variation in diagnostic procedures to test for GDM in Canada. As such there is a possibility that jurisdictions or institutions using a one-step approach will show a higher prevalence of GDM than those who opt for the two-step approach. While the ICD-10-CA coding is likely accurate, jurisdictional variations can exist due to testing practices rather than true differences in prevalence.

We noted some striking jurisdictional differences in our data for T1DM and T2DM. The highest prevalence of T1DM was found in Atlantic Canada, with the highest rates seen in Newfoundland and Labrador (1.75 times the Canadian rate). While there is considerable research suggesting that genetics are an established risk factor for the onset of T1, links to obesity, infection and environmental factors are still being explored [38]. Manitoba had over twice the prevalence of T2DM compared to the Canadian rate. It is unknown what may be driving the regional differences, and further research is needed.

There were limitations to this study. Our analyses do not contain Quebec data or data from individuals who choose to deliver at home. Small cell counts in the Northern Territories did not allow us to assess changes in rates over three time periods. No individual-level information such as weight, BMI, ethnicity, smoking status, family history, education or other socio-demographic information is available for analysis. These factors would be important to establish a profile of risk factors to help understand the key drivers among the population or to explore what may be contributing to regional differences.

Our study also has considerable strengths, which include a large population-based dataset, which contains almost all hospital deliveries in Canada excluding Quebec, and includes clinical indicators on the delivery, the health status of the person giving birth and infant outcomes. The data used in this study are of high quality and undergo strict data quality assurance processes. Furthermore, previous studies have shown that using ICD-10-CA codes to identify both T1DM and T2DM and GDM are valid, reliable, and accurate way to measure DM during pregnancy. As such, we expect that our findings represent a true approximation of prevalence in Canada.

## Conclusion

These Canadian data showed a steady increase in T1DM, T2DM and GDM among pregnancies in Canada. Continued national surveillance of DM during pregnancy is needed to better inform and guide prevention efforts, as the rates of T1DM and T2DM and GDM are expected to continue to rise due to the increased trend of an older, and more obese maternal population in Canada. Additionally, the increase in DM during pregnancy amongst the younger population warrants particular attention.

### Electronic supplementary material

Below is the link to the electronic supplementary material.

Supplementary Material 1

## Data Availability

The data that support the findings of this study were made available from the Canadian Institute for Health Information to fulfil the surveillance mandate of the Public Health Agency of Canada. The datasets analysed during the current study are not publicly available as they were provided by a third party through a data sharing agreement. Applications for accessing the data can be made by completing a request form through the Canadian Institute for Health Information’s website https://www.cihi.ca/en/data-inquiry-form Procedures to obtain data can be made available after making a reasonable request to the corresponding author.

## Abbreviations

DM: Diabetes mellitus
T1DM: Type 1 diabetes mellitus
T2DM: Type 2 diabetes mellitus
GDM: Gestational diabetes mellitus
CPSS: Canadian Perinatal Surveillance System
CIHI: Canadian Institute for Health Information
DAD: Discharge Abstract Database
LGA: Large for gestational age birthweight
SGA: Small for gestational age birthweight
CI: Confidence interval
SOGC: Society of Obstetricians and Gynaecologists of Canada
