# Supplementary material for: Diabetes mellitus in pregnancy across Canada
Source: BMC Pregnancy Childbirth. 2024 May 7;24:349. doi: 10.1186/s12884-024-06534-8 (PMC11075222; doi:10.1186/s12884-024-06534-8)
Supplement: Supplementary file 1 — Supplementary Material 1 [file 12884_2024_6534_MOESM1_ESM.docx]

**Supplemental Table 1. List of ICD-10-CA and CCI-10-CA codes used to identify the study population and select outcome**

| **Population or outcome** | **ICD-10-CA or CCI-10-CA code(s)** |
| --- | --- |
| Hospital delivery | Z37 or O10-O16, O21-O29, O30-O37, O40-O46, O48, O60-O75, O85-O92, O95 or O98-O99 with a ‘1’ or ‘2’ coded at the 6th digit |
| Caesarean delivery | 5MD60^^ |
| Induction of labour | 5AC30^^ |
| Gestational diabetes mellitus | O244—between 2005-2007  O248—from 2008 onwards |
| Type 1 diabetes mellitus | O240—between 2005-2007  O245—from 2008 onwards  E10 |
| Type 2 diabetes mellitus | O241—between 2005-2007  O246—from 2008 onwards  E11 |
| Singleton or multiple pregnancy | Singleton birth: Z370 (livebirth), Z371 (stillbirth)  Multiple birth: Z372, Z375 (livebirths), Z373, Z374, Z376, Z377 (stillbirths), Z3790 (unspecified) |
| Major congenital anomaly | Q00, Q01, Q05 if not Q00.0, Q03,Q04.1, Q04.2, Q11.0, Q11.1, Q11.2, Q16.0, Q17.2, Q20.0, Q20.1, Q20.3, Q20.5, Q21.2, Q21.3, Q23.4, Q25.1, Q30.0, Q35 (excluding Q35.7)  Q36, Q37, Q39.0-Q39.4, Q41, Q42.0-Q42.3  Q43.1, Q44.2, Q60.0-Q60.2, Q61.1-Q61.5, Q61.8, Q61.9, Q64.1, Q64.2, Q64.3, Q79.0, Q79.2, Q79.3, Q90, Q91.0-Q91.3, Q91.4-Q91.7, Q96 |
